# The association between health literacy and psychosomatic symptoms of adolescents in China: a cross-sectional study

**DOI:** 10.1186/s12889-019-7589-0

**Published:** 2019-09-12

**Authors:** Shi-chen Zhang, Dan-lin Li, Rong Yang, Yu-hui Wan, Fang-biao Tao, Jun Fang

**Affiliations:** 1 Department of Maternal and Child Health, School of Public Health, Anhui Medical University, 81th Meishan Road, 230032 Hefei, Anhui Province People’s Republic of China; 2 MOE Key Laboratory of Population Health Across Life Cycle / Anhui Provincial Key Laboratory of Population Health & Aristogenics, 81th Meishan Road, 230032 Hefei, Anhui Province People’s Republic of China; 3 Department of Toxicology, School of Public Health, Anhui Medical University, 81th Meishan Road, 230032 Hefei, Anhui Province People’s Republic of China; 4 Faculty of Pharmaceutical Science, Sojo University, Ikeda 4-22-1, Kumamoto, 860-0082 Japan

**Keywords:** Health literacy, Students, Psychosomatic symptoms

## Abstract

**Background:**

Lower health literacy (HL) has been known to be involved in a range of common mental and physical disorders among adolescent students. Ample studies indicated low HL is associated with a series of chronic diseases even psychological diseases, nevertheless, little is known about this relationship among adolescents. In this context, the study aimed to examine associations between psychosomatic symptoms (physical and psychological symptoms) and HL in junior and senior high school students in China, and to provide guidance for improving the physical and mental health in Chinese adolescents.

**Methods:**

A total of 22,628 junior and high school students in China were enrolled in this study. HL and psychosomatic symptoms were measured by self-report validated questionnaires. Multiple linear regression analyses were conducted to examine the associations between six sub-scales of HL and physical / psychological symptoms.

**Results:**

Multiple linear regression analysis demonstrated that the sub-scales of HL showed a significantly negative association with physical symptoms and psychological symptoms (*P* <  0.05 for each). Physical symptoms was most strongly associated with IR (*β* = − 0.134), followed by SM (*β* = − 0.093), DB (*β* = − 0.059), SA (*β* = − 0.058) and PA (*β* = − 0.054). No statistically significant difference was found between HA and physical symptoms (*P* > 0.05). Meanwhile, psychological symptoms were most strongly associated with IR (*β* = − 0.160), followed by SA (*β* = − 0.129), SM (*β* = − 0.069), DB (*β* = − 0.031), HA (*β* = − 0.026) and PA (*β* = − 0.021).

**Conclusion:**

These results indicated the importance of identifying the association of HL with physical and psychological symptoms, and provided the evidence that lower HL may serve as a critical and independent risk factor for poor health outcomes. Meanwhile, to maintain students’ desirable healthy status public health efforts for enhancing their HL level are urgently needed in adolescents.

## Background

Health literacy (HL), as defined by Ratzan and Parker [1] and adopted by Healthy People 2010 [1, 2] as well as by the Institute of Medicine (IOM) in their 2004 report [3], is “the degree to which individuals can obtain, process, and understand the basic health information and services they need to make appropriate health decisions”. HL is dependent on individual and systemic factors, such as communication skills with others, culture, demands of the health care, and so on. Moreover, HL affects people’s ability, including engaging in self-care and chronic-disease management, etc. [4]. In addition to basic literacy skills, HL requires knowledge of health topics. People with limited HL often lack knowledge or have misinformation about the body as well as the nature and causes of disease [3, 5].

From a social-emotional developmental perspective, adolescence is a period of life that may pose special vulnerability to physical and psychological impact factors, such as confusion and greater psychological pressure, even this is the peak period for the onset of mental disorders [6–9]. However, because the emotion regulation mechanism has not yet formed, namely lacking the ability to cope with negative emotion, they are prone to many mental and behavioral problems, such as cut class, sub-optimal physical/psychological health status, self-harm, suicide and others. Ample studies indicated low HL is associated, both directly and indirectly, with a series of adverse health outcomes [10–14], particularly with increased incidence of chronic diseases even psychological diseases, such as diabetes [15, 16], asthma [17], cancer [18], depression [19, 20], and schizophrenia [21]. Meanwhile, lower HL has been known to be involved in a range of common mental and physical disorder among adolescent students [21–26].

Research in the field of HL started late in China. The 2013 Health Literacy Survey of Chinese residents tested adolescents of 15 to 24 years old, and the result indicated that only 9.39% had adequate HL [27]. Ran M et al. suggested that the inadequate HL may contribute to poorer quality of life (QOL) among junior middle school students in Chongqing, China [28]. Nevertheless, most of these studies are based on small populations with a focus on patients with chronic diseases [15, 28], the studies using large samples to investigate this relationship among adolescents are few. In this context, in the present study we focused on the Chinese adolescent students’ HL, and administered a questionnaire survey among junior and high school students in six cities, China. The aim of this study was to investigate whether lower HL would be an independent risk factor for poor health outcome in Chinese adolescent students, and to provide guidance for improving the physical and mental health in Chinese adolescents.

## Methods

### Data collection and sampling

This study was conducted from November 2015 to January 2016 and was approved by the Ethics Committee of Anhui Medical University (Mar.1, 2014; approval number 20140087). The study was performed in accordance with the Declaration of Helsinki. All selected subjects were informed of the purpose of the study and were assured confidentiality upon receipt of the questionnaire. Consent to participate in the study was recorded in a separate consent form with the questionnaire, and it was confirmed upon completion and return of the questionnaire. This consent procedure was approved by ethics committees. Data were processed at a restricted location using a personal unidentifiable code for each subject.

Multistage stratified cluster sampling was conducted from junior and senior high schools located in 6 cities in China, including both urban and rural regions, as follows: Shenyang (capital of Liaoning Province), Xinxiang (north of Henan Province), Bengbu (Northeastern of Anhui province), Chongqing (one of China’s four direct-controlled municipalities), Ulanchap (Central Inner Mongolia Autonomous Region) and Yangjiang (Southwest coast of Guangdong Province). These cities are representative cities in China in terms of economic development and demographic composition, with average population, and they are also where our adolescent health research network is located. Eight schools in each city were selected including one key junior school, one ordinary junior school, one provincial key senior high school, one municipal key senior high school from rural regions and two ordinary junior schools, one ordinary senior high school and one municipal key senior high school from urban regions. Four to six classes were selected from each grade among grade 7 to 12 for investigation.

Approvals from schools, parents and students were obtained before carrying out this survey. Signed informed consent was requested to each participating school, and the students were allowed to participate in the study upon receiving completed written consent form from their parents. The team members explained the purposes and procedures of the study to the students, and provided an opportunity for them to ask questions. The students were allowed to withdraw from the study if they were not willing to participate. Under the supervision of teachers, each participant completed a self-report questionnaire, during 20–30 min in the classroom.

A total of 23,835 students were recruited in this study, participants were from grade 7–12 in junior and senior schools. The mean age of the participants was 15.36 (*SD* = 1.79). In the participating schools, 708 of the 23,835 sampled students were excluded from the study because of their absence from school on the day of the survey or reluctance to respond to the questionnaires. Thus, resulting in the receipt of 23,127 (97.0%) questionnaires, 22,628 (97.8%) of which were valid (questionnaire with missing data of > 5% were excluded).

### Design of questionnaires

The questionnaires consist of questions from demographic variables (i.e., gender, grade, registered residence, only child in family, self-reported family economy, father’s educational level, mother’s educational level, boarding on school days and support from friends), and the Multidimensional Sub-health Questionnaire of Adolescents (MSQA) and the Chinese Adolescent Interactive Health Literacy Questionnaire (CAIHLQ), as described below.

#### Instruments

MSQA which is developed by Tao et al. [29], with high reliability and validity, to evaluate the physical and psychological symptoms, was used in this study. The MSQA contains the following six sub-scales of two domains with total of 71 items: physical symptoms [physical inactivity (11 items), e.g. ‘Often feel sleepy and weak’; physical dysfunction (13 items), e.g. ‘Often feel a stomachache’; immunity decline (8 items), e.g. ‘Recurrent angular cheilitis’] and psychological symptoms [emotional symptoms (17 items), including depression and anxiety symptoms; conduct symptoms (9 items), including paranoid and hostile behaviors; and social adaptation symptoms (13 items), including bad relationships with family and friends]. Each question had 2 selection categories: 1 = no or last less than 1 month, 2 = lasts for more than 1 month and more. In the data analysis, no symptom and the symptom duration time < 1 month was assigned to 0 and the others were assigned to 1. The higher the score, the longer the symptoms lasted and the more serious the physical and psychological symptoms. The MSQA has been reported by various groups to be a valid and reliable method to explore determinants and the current state of Chinese adolescents’ psychosomatic health status [30–35]. In this study, the Cronbach’s α coefficient for the MSQA was 0.967, and 0.943 and 0.956 for physical and psychological symptoms.

We developed the Chinese Adolescent Interactive Health Literacy Questionnaire (CAIHLQ) [36], and used it to evaluate the adolescents’ HL level of this study. The CAIHLQ has been reported to be a valid and reliable measuring tool [37, 38]. The CAIHLQ consists of 31 questions grouped into 6 domains, as follows: (1) Physical activities (PA) of 6 items (e.g. ‘Following a planned exercise program.’); (2) Interpersonal relationship (IR) of 5 items (e.g. ‘Taking times with your family or friends.’); (3) Stress management (SM) of 6 items (e.g. ‘Balance time between study and play.’); (4) Self- actualization (SA) of 4 items (e.g. ‘Feeling each day is very meaningful.’); (5) Health awareness (HA) of 5 items (e.g. ‘Constricting sugars and food containing sugar.’); and (6) Dietary behavior (DB) of 5 items (e.g. ‘Eating 200-400 g of fresh fruit each day.’). Each item is rated on 5 selection categories (precontemplation, contemplation, preparation, action, and maintenance), and the total score is standardly converted to a score that ranges from 31 to 155, with lower scores indicating inadequate HL. In this study, internal consistency test showed that the Cronbach’s α coefficient was 0.910, and 0.662 to 0.847 for six sub-scales.

#### Statistical analysis

Statistical analysis was performed using SPSS ver. 23.0 for Windows (SPSS, Inc., Chicago, IL). The scores of HL were normally distributed and the variability in the data was homogeneous. The scores of physical and psychological symptoms were not normally distributed, so we performed logarithmic transformation. Cronbach’s alpha analysis was performed to determine the reliability of the survey. The Mann-Whitney test, Kruskal-Wallis H test, independent-Samples *t* Test and a one-way analysis of variance were used to assess group differences with respect to their statistical significance, while Bonferroni-adjusted *P*-value were calculated. The linear regression analysis was performed to examine the relationships between six sub-scales of HL and physical / psychological symptoms. Analyses were adjusted to control for key demographic and socioeconomic variables (i.e., gender, grade, registered residence, only child in family, self-reported family economy, parental education and support from friends). Statistical significance was set at *P* <  0.05.

## Results

### Univariate analysis

Table 1 presents the scores of the physical and psychological symptoms by general demographic characteristics. Senior high school students had significantly higher physical and psychological symptoms scores than junior school students (*Z* = − 14.091 and − 10.042, *P* <  0.001 for each), and higher physical and psychological symptoms scores were also found in students of boarding on school days, lower family income, none support from friends and more than one child in family (*P* <  0.001 for each). In addition, the results showed significant associations of gender (*Z* = − 2.625, *P =* 0.009), registered residence (*Z* = − 2.112, *P =* 0.035) and parental education (*Z* = -2.298 and − 2.623, *P* <  0.01 for each) with psychological symptoms; respondents whose parents had a higher education level had higher scores of psychological symptoms, and adolescents of female and urban had higher psychological symptoms level.

**Table 1:** Participant characteristics in the current study

Items	*n (%)*	Physical symptoms					Psychological symptoms				
		*M*	*P*_*25*_	*P*_*75*_	*χ*^*2*^/*Z*	*P-value* ^*a*^	*M*	*P*_*25*_	*P*_*75*_	*χ*^*2*^/*Z*	*P-value* ^*a*^
Gender					−0.188	0.851				−2.625	0.009
Male	10,990 (48.6)	1	0	3			2	0	9		
Female	11,638 (51.4)	0	0	4			2	0	9		
Grade					−14.091	< 0.001				−10.042	< 0.001
Junior school	11,993 (53.0)	0	0	3			2	0	8		
Senior high school	10,635 (47.0)	1	0	4			3	0	10		
Registered residence					−1.000	0.317				−2.112	0.035
Rural	10,882 (48.1)	0	0	4			2	0	9		
Urban	11,746 (51.9)	0	0	3			2	0	9		
Boarding on school days					13.647	< 0.001				10.570	< 0.001
Yes	11,320 (50.0)	1	0	4			3	0	10		
No	11,308 (50.0)	0	0	3			1	0	8		
Household structure					−5.575	< 0.001				−4.860	< 0.001
Only child	9720 (43.0)	1	0	4			2	0	10		
More than one child	12,908 (57.0)	0	0	3			2	0	9		
Father’s educational level					−0.626	0.531				−2.298	0.022
< High school degree	13,006 (57.5)	0	0	3			2	0	9		
≥ High school degree	9424 (41.6)	0	0	4			2	0	9		
No father	198 (0.9)										
Mother’s educational level					−1.230	0.219				−2.623	0.009
< High school degree	14,335 (63.4)	0	0	3			2	0	9		
≥ High school degree	8105 (35.8)	1	0	4			2	0	9		
No mother	188 (0.8)										
Self-reported family economy					308.706	< 0.001				355.664	< 0.001
Bad	3240 (14.3)	2	0	6			5	0	15		
General	16,345 (72.2)	0	0	3			2	0	8		
Good	3043 (13.4)	0	0	3			2	0	9		
Support from friends					257.148	< 0.001				444.541	< 0.001
None	599 (2.6)	3	0	13			12	2	24		
Few	14,535 (64.2)	1	0	4			2	0	10		
Lot	7494 (33.1)	0	0	3			1	0	7		

Statistical methods: *Mann-Whitney test or Kruskal- Wallis H test*. ^*a*^ is Bonferroni-adjusted *P*-value

The overall CAIHLQ mean score for all participants was 104.06 ± 18.68, among the six sub-scales of HL, the highest mean score was for SM (21.18 ± 5.16) and IR (20.73 ± 3.75), and the lowest mean score was for SA (15.02 ± 3.62) and HA (14.97 ± 4.39). As shown in Table 2, the students of junior school, urban, non-boarding on school days, higher parental education level, higher family income and more support from friends had higher scores of total HL and six sub-scales than the other groups (*P* <  0.001for each). Only child in family of students had higher scores of the sub-scales PA, SM, HA, DB and overall HL than non-one child students (*P* <  0.01 for each). Male students had higher scores of the sub-scales PA and DB than female, while the scores of the sub-scales IR and HA were lower than female (*P* <  0.001 for each). No significant differences were found in total HL and others sub-scales by gender (Table 2).

**Table 2:** Distribution of average score of HL according to CAIHLQ ^a^ based on completed and returned survey questionnaires

Variables (*n*)	PA	IR	SM	SA	HA	DB	Overall HL
Gender							
Male (10990)	15.89 ± 4.93	20.27 ± 3.97	21.18 ± 5.35	15.06 ± 3.72	14.65 ± 4.56	16.88 ± 4.25	103.94 ± 19.83
Female (11638)	15.04 ± 4.36	21.17 ± 3.47	21.17 ± 4.97	14.98 ± 3.54	15.27 ± 4.22	16.55 ± 3.40	104.17 ± 17.53
*t*	13.881	−18.194	0.168	1.695	−10.585	6.090	− 0.936
*P-value*	< 0.001	< 0.001	0.860	0.090	< 0.001	< 0.001	0.349
Grade							
Junior school (11993)	16.11 ± 4.70	20.91 ± 3.72	21.79 ± 5.21	15.36 ± 3.62	15.16 ± 4.43	17.10 ± 4.23	106.45 ± 18.94
Senior high school (10635)	14.71 ± 4.52	20.53 ± 3.76	20.49 ± 5.01	14.63 ± 3.61	14.74 ± 4.35	16.27 ± 3.95	101.37 ± 18.01
*t*	22.844	7.678	19.206	15.180	7.182	15.210	20.598
*P-value*	< 0.001	< 0.001	< 0.001	< 0.001	< 0.001	< 0.001	< 0.001
Registered residence							
Rural (10882)	15.08 ± 4.47	20.65 ± 3.71	20.85 ± 5.10	14.87 ± 3.61	14.74 ± 4.33	16.19 ± 4.10	102.38 ± 18.15
Urban (11746)	15.80 ± 4.81	20.81 ± 3.78	21.48 ± 5.20	15.16 ± 3.65	15.17 ± 4.45	17.19 ± 4.10	105.62 ± 19.03
*t*	−11.738	−3.386	−9.232	−5.967	− 7.373	− 18.209	−13.081
*P-value*	< 0.001	0.001	< 0.001	< 0.001	< 0.001	< 0.001	< 0.001
Boarding on school days							
Yes (11320)	14.84 ± 4.39	20.63 ± 3.66	20.69 ± 5.00	14.83 ± 3.55	14.71 ± 4.32	16.21 ± 4.00	101.90 ± 17.81
No (11308)	16.07 ± 4.84	20.84 ± 3.83	21.67 ± 5.27	15.22 ± 3.70	15.22 ± 4.46	17.21 ± 4.18	106.23 ± 19.27
*t*	−20.070	−4.202	−14.231	− 8.091	− 8.853	− 18.417	− 17.530
*P-value*	< 0.001	< 0.001	< 0.001	< 0.001	< 0.001	< 0.001	< 0.001
Household structure							
Only child (9720)	15.60 ± 4.79	20.72 ± 3.89	21.31 ± 5.23	15.03 ± 3.69	15.06 ± 4.58	17.11 ± 4.15	104.81 ± 19.20
More than one child (12908)	15.34 ± 4.57	20.75 ± 3.63	21.08 ± 5.10	15.02 ± 3.59	14.90 ± 4.35	16.41 ± 4.09	103.49 ± 18.26
*t*	4.131	− 0.596	3.332	0.141	2.658	12.624	5.266
*P-value*	< 0.001	0.551	0.001	0.888	0.008	< 0.001	< 0.001
Father’s educational level							
< High school degree (13006)	14.99 ± 4.41	20.59 ± 3.71	20.83 ± 5.08	14.80 ± 3.61	14.70 ± 4.31	16.29 ± 4.06	102.20 ± 18.05
≥ High school degree (9424)	16.10 ± 4.90	20.96 ± 3.75	21.68 ± 5.20	15.34 ± 3.61	15.35 ± 4.48	17.30 ± 4.11	106.73 ± 19.01
No father (198)	–	–	–	–	–	–	–
*t*	−17.695	−7.321	− 12.180	− 11.022	− 11.079	− 18.256	− 18.116
*P-value*	< 0.001	< 0.001	< 0.001	< 0.001	< 0.001	< 0.001	< 0.001
Mother’s educational level							
< High school degree (14335)	14.98 ± 4.41	20.58 ± 3.68	20.85 ± 5.06	14.80 ± 3.60	14.68 ± 4.31	16.34 ± 4.07	102.24 ± 17.94
≥ High school degree (8105)	16.30 ± 4.96	21.04 ± 3.79	21.79 ± 5.24	15.42 ± 3.63	15.48 ± 4.50	17.39 ± 4.12	107.42 ± 19.27
No mother (188)	–	–	–	–	–	–	–
*t*	−20.544	−8.805	− 13.270	−12.247	− 13.147	− 18.568	− 20.244
*P-value*	< 0.001	< 0.001	< 0.001	< 0.001	< 0.001	< 0.001	< 0.001
Self-reported family economy							
Bad (3240)	14.78 ± 4.69	19.52 ± 4.32	19.70 ± 5.40	14.08 ± 3.96	14.30 ± 4.53	15.79 ± 4.30	98.17 ± 19.99
General (16345)	15.35 ± 4.52	20.87 ± 3.55	21.26 ± 5.01	15.06 ± 3.52	14.97 ± 4.31	16.71 ± 4.02	104.23 ± 17.83
Good (3043)	16.70 ± 5.19	21.30 ± 3.82	22.33 ± 5.31	15.80 ± 3.64	15.63 ± 4.60	17.66 ± 4.28	109.43 ± 19.90
*F*	147.758	218.954	216.155	182.200	72.745	163.181	294.662
*P-value* ^*a*^	< 0.001	< 0.001	< 0.001	< 0.001	< 0.001	< 0.001	< 0.001
Support from friends							
None (599)	14.13 ± 5.77	16.21 ± 5.54	18.13 ± 6.37	12.66 ± 4.72	13.87 ± 5.06	15.06 ± 5.11	90.05 ± 20.01
Few (14535)	14.94 ± 4.37	20.37 ± 3.64	20.67 ± 5.02	14.66 ± 3.56	14.77 ± 4.31	16.45 ± 4.01	101.86 ± 17.74
Lot (7494)	16.56 ± 4.92	21.79 ± 3.35	22.42 ± 5.05	15.92 ± 3.46	15.43 ± 4.48	17.34 ± 4.18	109.45 ± 18.43
*F*	331.936	867.191	406.191	447.278	74.106	165.928	613.364
*P-value* ^*a*^	< 0.001	< 0.001	< 0.001	< 0.001	< 0.001	< 0.001	< 0.001

Statistical methods: Independent-Samples *t* Test or One-Way ANOVA

^*a*^ CAIHLQ, Chinese Adolescent Interactive Health Literacy Questionnaire. Overall HL is the mean score of all 31 items in the six subscales: PA is Physical Activity; IR is Interpersonal Relationships; SM is Stress Management; SA is Self-actualization; HA is Health awareness and DB is Dietary behavior. ^*a*^ is Bonferroni-adjusted *P*-value

### Multiple linear regression analysis

The total scores of psychosomatic symptoms according to MSQA were abnormally distributed, so we had a logarithmic transformation of the total score. We verified that evaluation of HL by CAIHLQ was indeed related to physical / psychological symptoms. This was determined from a single correlation analysis showing that overall scores of HL (*r* = − 0.218), PA (*r* = − 0.069), IR (*r* = − 0.221), SM (*r* = − 0.202), SA (*r* = − 0.190), HA (*r* = − 0.107) and DB (*r* = − 0.141) were significantly correlated with physical symptoms. Moreover, results from correlation analyses revealed that overall scores of HL (*r* = − 0.287), PA (*r* = − 0.120), IR (*r* = − 0.282), SM (*r* = − 0.241), SA (*r* = − 0.271), HA (*r* = − 0.155) and DB (*r* = − 0.143) were significantly correlated with psychological symptoms (*P* <  0.001 for each, Table 3).

**Table 3:** Correlations between HL with both physical symptoms and psychological symptoms for participants

	Overall HL	PA	IR	SM	SA	HA	DB	Physical symptoms	Psychological symptoms
Overall HL	–								
PA	0.692^***^	–							
IR	0.688^***^	0.313^***^	–						
SM	0.823^***^	0.448^***^	0.567^***^	–					
SA	0.772^***^	0.416^***^	0.346^***^	0.634^***^	–				
HA	0.723^***^	0.429^***^	0.314^***^	0.491^***^	0.453^***^	–			
DB	0.641^***^	0.336^***^	0.688^***^	0.401^***^	0.354^***^	0.395^***^	–		
Physical symptoms	−0.218^***^	−0.069^***^	−0.221^***^	− 0.202^***^	− 0.190^***^	− 0.107^***^	−0.141^***^	–	
Psychological symptoms	−0.287^***^	− 0.120^***^	− 0.282^***^	− 0.241^***^	−0.271^***^	− 0.155^***^	−0.143^***^	0.518^***^	–

Overall HL is the mean score of all 31 items in the six subscales: PA is Physical Activity; IR is Interpersonal Relationships; SM is Stress Management; SA is Self-actualization; HA is Health awareness and DB is Dietary behavior.^***^ *P* < 0.001

We further carried out multiple linear regression analysis adjusted for key demographic variables (e.g. support from friends, household structure, boarding on school days, self-reported family economy, gender and grade), using logarithmic transformation of total scores of physical / psychological symptoms as a dependent variable, and overall and the six sub-scales of HL as independent variables, respectively. The total score and scores of six sub-scales exist in a collinear manner, Table 4 showed the correlation between six sub-scales and physical / psychological symptoms. The models fitted well (*P* <  0.001 for each model).

**Table 4:** Multiple linear regression for psychosomatic symptoms according to health literacy

Variable	Physical symptoms ^*a*^				Psychological symptoms ^*b*^			
	*B (SE)*	*95%CI*	*β*	*P-value*	*B (SE)*	*95%CI*	*β*	*P-value*
PA	−0.005 (0.001)	− 0.007, − 0.003	−0.054	< 0.001	− 0.002 (0.001)	− 0.004, − 0.001	−0.021	0.024
IR	−0.015 (0.001)	−0.017, − 0.012	−0.134	< 0.001	− 0.019 (0.001)	− 0.022, − 0.017	−0.160	< 0.001
SM	−0.008 (0.001)	−0.010, − 0.006	−0.093	< 0.001	− 0.006 (0.001)	− 0.008, − 0.004	−0.069	< 0.001
SA	−0.007 (0.001)	−0.009, − 0.004	−0.058	< 0.001	− 0.016 (0.001)	− 0.019, − 0.014	−0.129	< 0.001
HA	0.001 (0.001)	0.002, 0.003	0.001	0.893	−0.003 (0.001)	−0.005, − 0.001	−0.026	< 0.001
DB	−0.006 (0.001)	−0.008, − 0.004	−0.059	< 0.001	− 0.004 (0.001)	− 0.006, − 0.002	−0.031	0.006
*⊿R* ^*2*^	0.071^***^					0.110		

^*a*^ Adjusted for grade, boarding on school days, household structure, self-reported family economy and support from friends; ^*b*^ Adjusted for gender, grade, registered residence, boarding on school days, household structure, parents’ educational level, self-reported family economy and support from friends.PA is Physical Activity; IR is Interpersonal Relationships; SM is Stress Management; SA is Self-actualization; HA is Health awareness and DB is Dietary behavior.*⊿R*^*2*^ is coefficient of determination

As shown in Table 4, the sub-scales of HL showed a significantly negative association with physical symptoms and psychological symptoms (*P* <  0.05 for each). Physical symptoms were most strongly associated with IR (*β* = − 0.134), followed by SM (*β* = − 0.093), DB (*β* = − 0.059), SA (*β* = − 0.058) and PA (*β* = − 0.054). No statistically significant difference was found between HA and physical symptoms (*P* > 0.05). Meanwhile, psychological symptoms were most strongly associated with IR (*β* = − 0.160), followed by SA (*β* = − 0.129), SM (*β* = − 0.069), DB (*β* = − 0.031), HA (*β* = − 0.026) and PA (*β* = − 0.021).

## Discussion

This study is focused on the Chinese adolescent students’ HL, examining the associations between psychosomatic symptoms and HL in junior and high school students in China. As hypothesized, compared with students with higher HL, those with lower HL have shown more physical and psychological symptoms. Associations remained significant after controlling for demographics. Thus, HL appears to be independently associated with certain known predictors of poor health status. Individuals with lower HL might be inclined to have mental disorders and physical illness.

Our findings indicated that the students of junior school, urban, non-boarding on school days, higher parental education level, higher family income and more support from friends had higher scores of total HL and six sub-scales than the other groups, these findings are congruent with results from the previous studies [27, 37, 39]. In the current study, the students of lower family income, none support from friends and non-one child in family had significantly higher physical and psychological symptoms scores, consistent with other studies. It is indicated that parental companionship plays an important role in adolescent health development [30–33]. At the same time, parents in better financial condition will have more time and energy to communicate and support with their children, thus reducing the occurrence and development of their children’s physical and psychological problems [40, 41].

We found that lower student’s HL is associated with higher odds of poor health outcome, even after adjustment for the influence of demographics. Physical symptoms and psychological symptoms were shown to have similar tendencies on the scores of six sub-scales, as well as in the correlations among variables (Tables 3 and 4). The items of six sub-scales are recognized as advantageous factors for achieving a high QOL, by reducing stress and by enhancing relationships, respectively [42].

In this study, the six sub-scales of HL were significantly associated with each factor, showing IR have the highest *β*-value, followed by SM, DB, SA and PA. Similar results were found in both physical and psychological symptoms. The interpersonal relationships have been linked not only to mental health but also to morbidity and mortality [43, 44], which is consistent with the current study. Accordingly, interpersonal relationships facilitate healthier behaviors and adherence to medical regimens, which in turn protect the subject from developing the disease [45]. Additionally, other potential mechanisms that might explain how social support can health are related to biological mechanisms, especially the immune-mediated inflammatory processes [46]. Petersen S et al. reported that recurrent pain symptoms in children, particularly frequent symptoms, should be regarded as a potential general pain disorder rather than merely a localized body disorder, they also reflected psychological stress [47]. Therefore, students with low levels of SM, IR and SA have increased psychological stresses and negative emotions, which will increase the incidence of pain-related items in physical symptoms [48, 49].

Although the mechanism between HL and health outcomes has not been clarified, Bailey SC et al. found through path analysis that HL influences self-efficacy through knowledge, and then directly or indirectly (through physical activity) influences health outcomes [50]. The correlation between the sub-scales of PA and physical and psychological symptoms may be realized through this path in this study. A growing body of evidence indicates that PA can have beneficial effects on physical and mental health in adolescents [51]. The odds for depressive symptoms were lower for those who were physically active in a sports club (*OR*: 0.40, 95% *CI*: 0.30–0.53) [52]. It suggests a possible role of social interaction in addition to physical activity per se [52]. Unhealthy dietary behavior (e.g. irregularity of meals and breakfast skipping, etc.) play an important role in developing physical and mental health in adolescents [53]. It has been showed that the association between stress and unhealthy dietary behavior may also be mediated by avoidant coping, and that stress may affect health in an indirect way through unhealthy dietary behaviors [54]. Though the cross-sectional nature of the study limits causal inference, it strongly suggested that interventions for improving adolescent student’s HL will reducing their physical and psychological symptoms.

HA is one of the major indicators which reveal a person’s knowledge about health problems [55]. In this study, no statistically significant difference was found between HA and physical symptoms, which is worth emphasizing. These students did not feel any desirable degree of health awareness probably because young people do not consider health controls as being necessary issue relating to healthy life in China. Generally, if the individual does not realize his own health problems, he will not make any effort to improve his health [56]. It is thus particularly important for adolescents to not only promote their health, but also have a suitable health awareness [57].

In this study, we selected the participants from both rural and urban regions, considering the difference in the different socioeconomic conditions. Nevertheless, only six cities were surveyed, the validity of this study for students in other regions, countries or cultures is not fully clear and need further investigations. We suggest further research with larger study populations and in different regions of the country. It should also be noted that the reliance on self-report measures is a possible limitation in this study, which may be biased in the measuring process. Moreover, another limitation is that both the independent variable and dependent variable are constructs that cannot be measured precisely. Thus, latent variable methods should be applied in the future to account properly for the latent nature of those constructs. Furthermore, the missing students were not included in the survey, and these students may have a lower HL and may have more psychosomatic symptoms. In addition, because the analyses in this study used cross-sectional data, the results may not imply causality but rather demonstrate an independent association between HL and poor health outcomes. Longitudinal studies will be used to clarity temporal relationship of HL to physical/psychological symptoms in the future study.

## Conclusion

Taken together, our results indicated the importance of identifying the correlates of HL and psychosomatic symptoms, and provided the evidence that HL may serve as a critical and independent risk factor for poor health outcomes. Meanwhile, health guidance and intervention program for adolescent students from school, family and community to enhance their health literacy levels would be effective measures to maintain a desirable healthy status. Additionally, it merits further longitudinal studies to confirm the impact of HL on psychosomatic symptoms, and verify the hypothesis that improving students HL may be effective in modifying psychosomatic symptoms.

## Data Availability

We could not provide data set publicly because of the ethical restrictions and privacy concerns. However, those data will be available upon request to all interested researchers, in this case, please contact the Ethics Committee of Anhui Medical University (+ 86–551-65161057).

## Abbreviations

CAIHLQ: Chinese Adolescent Interactive Health Literacy Questionnaire
DB: Dietary behavior
HA: Health awareness
HL: health literacy
IR: Interpersonal relationship
MSQA: Multidimensional Sub-health Questionnaire of Adolescents
PA: Physical activities
SA: Self – actualization
SM: Stress management
